# Null regions: a unified conceptual framework for statistical inference

**DOI:** 10.1098/rsos.221328

**Published:** 2023-11-22

**Authors:** Adam H. Smiley, Jessica J. Glazier, Yuichi Shoda

**Affiliations:** ^1^ Department of Psychology, University of Washington, Seattle, WA 98195, USA; ^2^ Department of Psychological Science & Neuroscience, Belmont University, Nashville, TN 37212, USA; ^3^ Department of Psychology, Northeastern University, Boston, MA 02115, USA

**Keywords:** clinical and practical significance, minimum-effect testing, equivalence testing, strong form hypothesis testing, non-inferiority testing, open science

## Abstract

Ruling out the possibility that there is absolutely no effect or association between variables may be a good first step, but it is rarely the ultimate goal of science. Yet that is the only inference provided by traditional null hypothesis significance testing (NHST), which has been a mainstay of many scientific fields. Reliance on NHST also makes it difficult to define what it means to replicate a finding, and leads to an uncomfortable quandary in which increasing precision in data reduces researchers' ability to perform theory falsification. To solve these problems, in recent years several alternatives to traditional NHST have been proposed. However, each new test is described using its own terminology and practiced in different fields. We describe a simple, unified framework for conceptualizing all these tests so that it is not necessary to learn them separately. Moreover, the framework allows researchers to conduct any of these tests by asking just one question: is the confidence interval entirely outside the *null region(s)*? This framework may also help researchers choose the test(s) that best answers their research question when simply ruling out ‘no effect at all’ is not enough.

## 1. Introduction

Traditional null hypothesis significance testing (NHST) answers only one question: can one, with reasonable confidence, rule out the counterfactual, ‘null’ hypothesis that there is no effect at all? To illustrate, suppose a study found that treatment A produced better results than treatment B, and that the difference was statistically significant. What exactly does it mean that the difference was statistically significant? It means that the difference observed in this study was unlikely to occur if the truth was that there is absolutely no difference. Ruling out that there is absolutely no difference may be a good first step, but it is rarely the ultimate goal of empirical research. Yet that is the only inference provided by traditional NHST, which has been a mainstay of many scientific fields.

Reliance on NHST also makes it difficult to define what it means to replicate a finding. Suppose a new study also found a statistically significant difference between treatments A and B. Should the new study be considered to have replicated the original study? What if the new study tested 10 times as many study participants as the original study? All things being equal, estimates based on a larger sample are more precise and thus make it easier to find statistically significant results (i.e. easier to rule out the possibility that in truth, there is absolutely no difference). Therefore, the difference found in the new study, although statistically significant, may be much smaller than what was found in the original study, almost to the point where it is of little consequence. This can occur with increasingly larger samples made available by ‘big data’. If the effect found in the new study can be deemed statistically significant even if it is too small to be of consequence, does it make sense to consider the original finding replicated? Yes, if we go by statistical significance. No, if we consider that the effect found in the new study was much smaller than in the original study. The fact that a larger study, with more accurate estimates, makes it easier to ‘replicate’ the original finding (if replication is defined by statistical significance) and considered as supporting the theory proposed to account for the original finding, leads to an uncomfortable quandary: increasing the precision in research reduces researchers' ability to perform theory falsification. In addition, if an original finding was published primarily because it was statistically significant, such use of traditional NHST to define a ‘finding’ can be considered one of the reasons ‘why most published findings are false’ [1].

While statistical significance by traditional NHST is not very informative (because it does not necessarily indicate that the findings are scientifically or practically significant), lack of statistical significance by the traditional NHST is not particularly informative either. Namely, failing to reject the null hypothesis of no effect does not allow one to infer that an effect does not exist*.* The effect may in fact exist, but the research may have failed to detect it due to low statistical power. In addition, NHST does not allow one to infer if a new treatment results in a large enough improvement over no treatment, or if a new, easier-to-administer treatment with fewer side effects is not substantially inferior to the current treatment with regard to a main outcome (e.g. survival).

Because traditional NHST only tests the null hypothesis that there is absolutely no effect – relying on it often means settling for a statistical test that does not adequately address the research question. It would be desirable to be able to test the null hypotheses that are more relevant to the research question (e.g. the true effect is too weak to matter; or that it is too far from the theoretically predicted effect).

To solve these problems, in recent years several alternatives to the traditional NHST have been proposed. These new alternatives share one common feature: they state the null hypothesis as a span of values, that is, the null hypothesis refers to a region, or regions, rather than a single point (of absolutely no effect). Yet because each new test is described using its own terminology and practiced in different scientific fields, few researchers are familiar with more than one or two of them, even though they all use the same underlying statistical framework. In this paper, we describe how all these tests can be conceptualized within a common framework so that it is not necessary to learn them separately. Moreover, the framework allows researchers to conduct each test by asking just one simple question: is the confidence interval entirely outside the null region(s)? We believe this framework provides a viable supplement to traditional NHST and would like to offer it for consideration by the scientific communities.

### 1.1. Null hypothesis significance testing and the search for an alternative

For some research questions, small effects may be of theoretical importance (e.g. [2,3]). However, in many circumstances it is misleading to refer to very small effects as *significant*, which in common usage means ‘sufficiently great or important to be worthy of attention; noteworthy; consequential, influential’ [4]. Meehl [5] argued that when using traditional NHST, increasing precision (e.g. larger sample size) virtually guarantees that a ‘statistically significant’ result can be found to support any theory even ‘if the theory is entirely without merit’^1^ (p. 111). That is, with NHST, increasing sample size will increase the likelihood of statistical significance even when the true effect is too small to matter, leading to a false impression that the effect is large enough to matter [7]. Adopting a criterion more stringent than the commonly used *p* < 0.05 (e.g. *p* < 0.005 [8]) would increase NHST's evidentiary strength (reflected in the relative odds of alternative versus null hypotheses, i.e. the Bayes factor), as well as the replicability of statistically significant results. However, even with a different standard, NHST remains fundamentally a test against the null hypothesis of no effect at all. As a result, it does not allow researchers to ask questions beyond ‘is there absolutely no effect?’ In fact, there are many other questions researchers may want to answer by conducting empirical studies, such as ‘is the effect strong enough to matter?’, ‘is it close enough to a theoretically predicted value?’, or ‘are the data not informative enough to draw any conclusion?’

With widely recognized problems associated with NHST such as those described above (see also [9]), many have advocated abandoning statistical significance testing altogether in favour of focusing primarily on confidence intervals (e.g. [10,11]). Confidence intervals contain much more information than NHST. However, in practice, researchers often use confidence intervals only to ask if they contain zero, which is functionally no different from traditional null hypothesis testing [12]. Furthermore, many researchers have ‘a gross misunderstanding’ of the meaning of confidence intervals [13] (see also [14]), such as interpreting confidence intervals as though they can answer the Bayesian question, ‘given the data, where is the true effect likely to be?’^2^

When used along with confidence intervals, discrete decision criteria can be useful. For example, research on decision making under uncertainty has found that focusing on a discrete consequential outcome while also describing its degree of uncertainty promotes the most effective use of uncertainty information (e.g. [16]). Discrete decision criterion is also useful for stating the results concisely (e.g. ‘X had a significant effect on Y’ is more concise than ‘the effect of X on Y is such-and-such, with a confidence interval of [lower bound, upper bound]’). Additionally, having discrete decision criteria is a necessary component of theory falsification; dichotomous claims can be supported or refuted by future evidence [17]. Authors and editors may also find it useful to have a discrete criterion for deciding what findings are reasonable to be mentioned in the abstract. Thus, the persistent use of NHST may indicate the heuristic value of a discrete decision criterion. Is there a way to preserve these benefits without suffering from the myriad problems of NHST?

Below we offer a framework that attempts to answer this question. One can conceptualize these tests within the same framework, each asking different questions and providing bases for different discrete decisions. It does so while keeping (or increasing) the focus on confidence intervals. Visualizing these different tests side-by-side, the framework makes it easier to see how they relate to each other, and facilitates choosing the test that most directly addresses each research question. All tests can be conducted by applying the same simple criterion (i.e. the confidence interval being entirely outside of the null region(s) specified by a given test), without needing to use different statistical software. In addition, when none of the multiple types of tests in this framework yield significant results, one has a stronger basis for concluding that the available data are not sufficient to make any inferences, an important message to convey in order to prevent an erroneous, and dangerous, impression of certainty (e.g. [18]). Below we briefly describe these tests, starting with minimum-effect testing, equivalence testing, strong form hypothesis testing, and non-inferiority testing.

### 1.2. Minimum-effect testing

Minimum-effect testing (MET) allows researchers to test if an effect is large enough to matter by testing if an observed value falls sufficiently *outside* of a range of values that are considered to be inconsequential [19]. For example, suppose that the results of a study seeking to replicate the original finding of treatment A being superior to treatment B found that A was still statistically significantly better than B by a traditional NHST (i.e. the null hypothesis of absolutely no difference could be rejected), but the difference between the two was much smaller than originally found. Should we consider the original finding as replicated? MET can facilitate answering this question by testing: is the effect (i.e. the difference between the treatments) found in the replication study large enough to warrant considering the original finding replicated?

Historically, building on the ‘good-enough principle’ advocated by Serlin & Lapsley [6], Murphy & Myors [19] developed MET using a version of an *F* test to determine if the observed effect is larger than can be reasonably expected from the pre-specified minimally consequential variance accounted for. However, in the over twenty years since Murphy and Myors' article was published, MET has been seldom used for empirical research. We suspect this is largely because of the difficulty associated with conducting MET with *F* tests, notwithstanding the workaround proposed by Fowler [20] as well as the lack of versatility offered by the particular method proposed by Murphy and Myors. However, as with all the other tests in the framework, MET can also be conducted by asking just one question: is the confidence interval entirely outside the *null region(s)*? This should make MET more accessible, as it can be applied to any statistic for which confidence intervals can be calculated (i.e. not just *F*).

**Figure 1.**
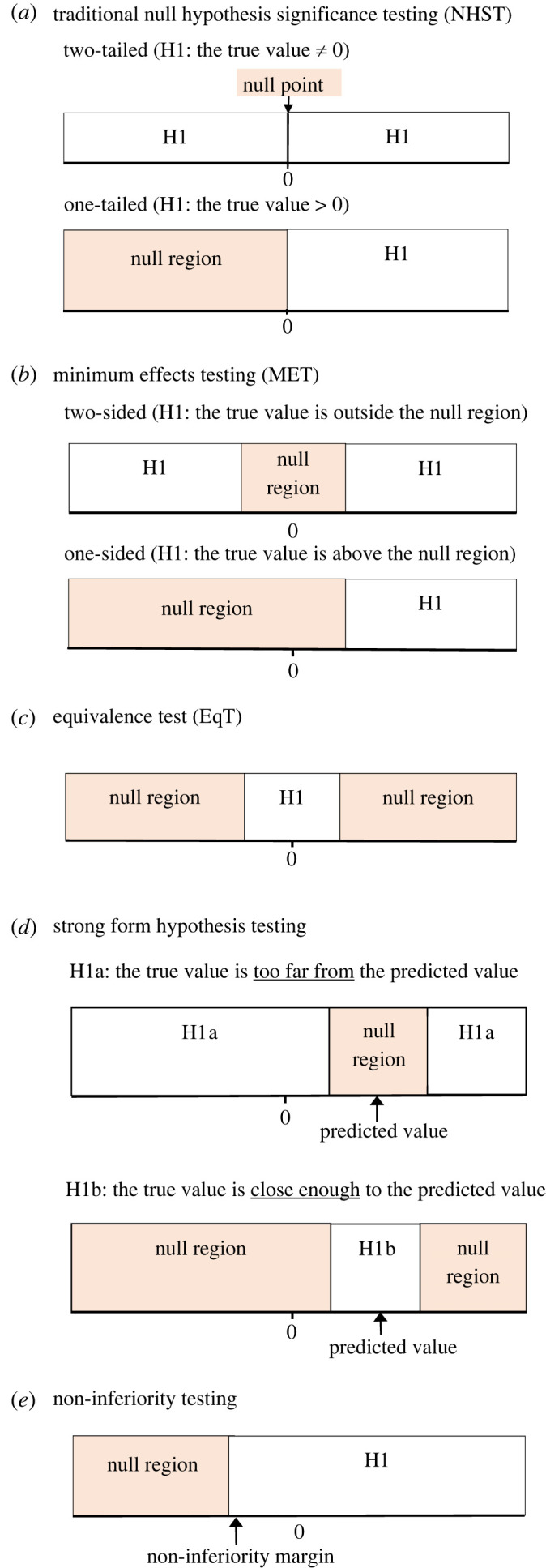
The null regions for MET, EqT, strong form hypothesis testing, and the non-inferiority test, in terms of true (i.e. population) effects. Null regions are the population values that each test seeks to rule out in favour of alternative hypotheses (H1). This figure uses the format used by fig. 1 of Lakens *et al*. [21].

MET solves one key shortcoming of NHST: with NHST, increasing sample size will increase the likelihood of statistical significance even when the true effect is too small to matter. With MET, as shown in figure 1 and detailed in supplementary material 1, if the observed effect is weaker than the smallest possible effect considered to be meaningful, MET will *never* be significant, regardless of sample size. On the other hand, if the observed effect is stronger than the smallest effect of consequence, the larger the sample size, the greater the likelihood that it is significant by MET. One-sided MET is analogous to one-sided NHST except that the null region extends beyond 0 to the weakest effect still considered meaningful.

### 1.3. Equivalence testing: more than ‘retaining the null’

Equivalence testing (EqT) examines whether an effect is so weak that it is practically equivalent to zero. To illustrate, suppose a new study found that the difference in the effectiveness of Treatment A and Treatment B was very small and not statistically significant by NHST. Based on this, can one infer that the effectiveness of the two treatments is the same for all practical purposes? Not necessarily, because traditional NHST may fail to be statistically significant due to low statistical power to detect differences, not because there is no meaningful difference. In this situation, researchers can use EqT to see if the findings warrant inferring that differences are so small as to be inconsequential in practice.

EqT overcomes another shortcoming of the traditional NHST. In the case of a non-significant result by NHST, researchers cannot rule out the possibility that there may be a true effect. However, by using EqT, researchers can infer, with reasonable confidence, that the true effect in the population is too small to be meaningful (e.g. [21,22]). EqT can also be conducted by asking just one question: is the confidence interval entirely outside the *null region(s)* [23–26]?

### 1.4. Strong form hypothesis testing

A central goal of empirical science is to find a theory that best explains and predicts observations of the world. We accomplish this by generating many theories and ruling out those that do not do as good a job as others (e.g. [27]). Increasing sample sizes and the associated increases in precision should facilitate this process by making it easier to reject imperfect theories. Yet, as Paul Meehl [7] once famously lamented, the opposite is the case when using the traditional form of NHST: the larger the sample size and the more precise the measurement, the more difficult it is to rule out *any* theory as long as it predicts a non-zero effect. To remedy this, Meehl suggested a ‘strong use of a significance test’ as originally suggested by Karl Pearson, such that ‘the more precise the experiment, the more dangerous for the theory’. Using the proposed null region framework, we propose what we refer to as ‘strong form hypothesis testing’ to examine whether observed values are in line with what is expected from a theory.

Suppose an original study found Treatment A was superior to Treatment B in 75% of the population. The authors of that study consider the results to be in support of the theory predicting exactly such an effect (e.g. the treatment effects reflect the population prevalence of a particular variant of a gene). Now consider that a study seeking to replicate the original finding found that Treatment A was superior to Treatment B in 60% of the population even though the theory predicted 75%. Does the new result support the theory, or does it show a shortcoming of the theory because the new finding did not show exactly the predicted effect? Should the theory be retained, or modified or replaced by a new theory? Strong form hypothesis testing is designed to answer this question.

Strong form hypothesis testing shown in figure 1 consists of two parts. The first part is conceptually similar to MET. While MET asks if the true effect is ‘large enough to matter’, Part 1 of strong form hypothesis testing asks if the true effect is ‘different enough from the theoretically predicted value’. The null hypothesis is that the true value is close enough to the theoretically predicted value. Thus a significant result rejecting this null hypothesis suggests that the theory be modified or replaced. Part 2 is conceptually similar to EqT. While EqT asks if the true effect is ‘close enough to zero’, Part 2 of strong form hypothesis testing asks if the true effect is ‘close enough to the theoretically predicted value’. The null hypothesis is that the true value is too far from the theoretically predicted. A significant result rejecting this null hypothesis therefore suggests that we retain the theory. In short, Part 1 asks ‘should we reject the theory?’ while Part 2 asks ‘should we retain the theory?’ If neither Part 1 nor Part 2 are significant, it suggests that more, and more precise, data are needed, because the current data do not provide sufficient information to warrant either inference, for or against, the theory. Similar to MET, EqT, and non-inferiority testing, both parts of the strong form hypothesis testing can be conducted by asking just one question: is the confidence interval entirely outside the *null region(s)*?

### 1.5. Non-inferiority testing

Sometimes, researchers—particularly those who conduct clinical trials of a new treatment—seek evidence that the new treatment is not substantially inferior to the treatment currently considered standard. For example, a new cancer treatment known to have fewer side effects than the standard treatment may still be preferable even if its beneficial effect is technically somewhat weaker than the standard treatment for the main outcome (e.g. cancer-free survival) as long as the difference is not clinically consequential. The traditional NHST is not suitable in this context. To illustrate, suppose traditional NHST found that there is no statistically significant difference between the new and the standard treatment. However, this does not provide the evidence needed, because non-significant NHST may simply be the result of insufficient statistical power even if, in truth, the new treatment is substantially inferior to the standard one. Alternatively, suppose traditional NHST found that the new treatment *is* statistically significantly worse than the standard treatment? Does that mean one should abandon the new treatment? Not necessarily, because the new treatment may only be trivially worse, in which case it may still be preferable because it has much less severe side effects. How can we test the null hypothesis that the new treatment is *good enough*, meaning that it is no more than trivially worse than the standard treatment?

Non-inferiority testing (e.g. [28]) answers this question, by testing the null hypothesis that the true effect of the new treatment is more than trivially worse than the standard treatment. Rejecting the null hypothesis supports the inference that the new treatment is for all practical purposes good enough, or ‘non-inferior’, and therefore it is preferable because of its other desirable qualities (e.g. fewer side effects). For example, using this test, a 2022 publication in *Lancet* comparing treatments for cancer concluded that ‘[t]he findings demonstrate that per-protocol treatment with imiquimod is non-inferior to surgical treatment’ [30, p.1795].

During the last two decades, non-inferiority testing has been widely used in clinical trials research. Non-inferiority testing was incorporated in the CONSORT standard for reporting clinical trials in 2006 [28], and a MEDLINE search has shown that non-inferiority testing was referenced in over 1500 publications in 2022 alone. While non-inferiority testing is most commonly used for clinical trials, it may be applicable to a wide range of research questions such as those comparing different methods for reducing carbon emission to mitigate climate change, or for reducing poverty and income disparities, and many more. Similar to MET, EqT, and strong form hypothesis testing, it can be conducted by asking just one question: is the confidence interval entirely outside the *null region*? When conducted this way using confidence intervals, none of these tests require any specialized statistical software whenever confidence intervals for the statistic of interest have been computed.

## 2. A unified framework using null regions

Because MET, EqT, strong form hypothesis testing, and non-inferiority testing use the same underlying logic, they can naturally be integrated into a common framework, providing researchers clear insights into how they relate to each other, and which test(s) is most relevant to their research question. We suspect often the most relevant test may not be the traditional NHST, and we hope the framework helps researchers see at a glance the range of tests from which to choose one (or more) that is/are most appropriate for their research question. This framework is shown in figure 1. In MET, researchers test if they can reject the null hypothesis that the true effect is within the range of values that are not strong enough to matter. EqT does the opposite, testing if they can reject the null hypothesis that the true effect is within the ranges of values that are either too positive or too negative to be considered equivalent to zero [21]. In Part 1 of strong form hypothesis testing, researchers test if they can reject the null hypothesis that the true effect is within the ranges of values that are consistent with the theoretically predicted value. Rejecting this null hypothesis suggests that the findings are inconsistent with the theory. Part 2 of strong form hypothesis testing tests the null hypothesis that the true effect is too far away from the theoretically predicted value. Rejecting this null hypothesis suggests that the findings are consistent with the theory. And in non-inferiority testing, researchers test if they can reject the null hypothesis that the true effect of a new treatment is within a range of values that are meaningfully worse than standard treatments. What all these tests have in common is that their respective null hypotheses all state that the true (i.e. population) value is within a certain range or ranges, in contrast to the traditional two-tailed NHST that states its null hypothesis in reference to a null point (of no effect at all). In order to facilitate the use of the common framework underlying these tests we propose referring to the range of values representing the null hypothesis with the same term (rather than a different one for each test). We propose calling them ‘null regions’, as shown in figure 1.

We believe figure 1 will facilitate the choice of the statistical test that is most appropriate to the research question being investigated by a study. Figure 1 shows how five different hypothesis tests (including the one-tailed version of the traditional NHST) can be conceptualized with regards to null regions. The null region(s), in pink, represents the range of *population parameters* which constitute the null hypothesis—that is to say, if it is plausible that the observed effect could be obtained even if the true effect falls within this range, the null hypothesis will be retained (i.e. the test is not significant). On the other hand, if the observed effect is unlikely if the true effect falls within the null region or regions then the null hypothesis is rejected (i.e. the test is significant). The regions labelled ‘H1’ represent the range of population parameters which constitute the alternative hypothesis that are supported when the null hypothesis is rejected.

Note, however, that just because the *observed effect* is within H1, that does not mean that we have sufficient evidence to reject the null hypothesis, as that could occur even if the true population value is within a null region. We describe the process of making this determination in the following section (Conducting significance tests).

Sometimes using more than one of these tests simultaneously can improve the informativeness of the study. For example, if MET was not significant, researchers may wonder if the data allow inferring that the effect is practically zero, or if it is not significant is because the data are not precise enough to allow any inferences. These two possibilities can be distinguished by conducting EqT in addition to MET, and the researchers can go beyond simply failing to reject the MET null hypothesis. In this scenario, if EqT is significant, then researchers can infer that the true effect is close enough to zero as to be considered ‘practically zero’ (i.e. we reject the null hypothesis that the true effect is *within* the null regions for EqT). The logic for the strong form hypothesis testing is the same, in effect combining EqT with MET, with null regions defined in reference to the theoretically predicted value. If none of the tests relevant to the research question of interest are significant, then researchers have a reasonable basis for concluding that the data *do not provide sufficient evidence* to support either inference (see [31]). This is important because recognizing and stating uncertainty is the bedrock of science and a driver of scientific progress (e.g. [32]).

### 2.1. Conducting significance tests

Since its inception two decades ago, non-inferiority testing has been conducted by asking one simple question: does the confidence interval, based on the observed data, fall entirely outside of the null region [28,29]? In the case of non-inferiority testing, the null region is any value below the ‘non-inferiority margin’, representing the values of unacceptably poor performance of the new treatment being tested compared against a standard treatment. As shown in electronic supplementary material S2, this criterion can also be used to conduct MET (as well as EqT). The proposed framework allows researchers to conduct any of the tests in this framework by applying the following simple decision rule: if the confidence interval based on the observed data falls *entirely outside* of the null region (or both null regions in the case of EqT or Part 1 of the strong form hypothesis testing) for a given test, then that test is significant. By contrast, if the CI is *partially or entirely within* a null region then the test is not significant. The confidence level (e.g. 95%) used for computing the width of the confidence interval is up to the user—this choice will depend on weighing the tradeoffs between statistical power and the rate of false positives (type I errors).

**Figure 2.**
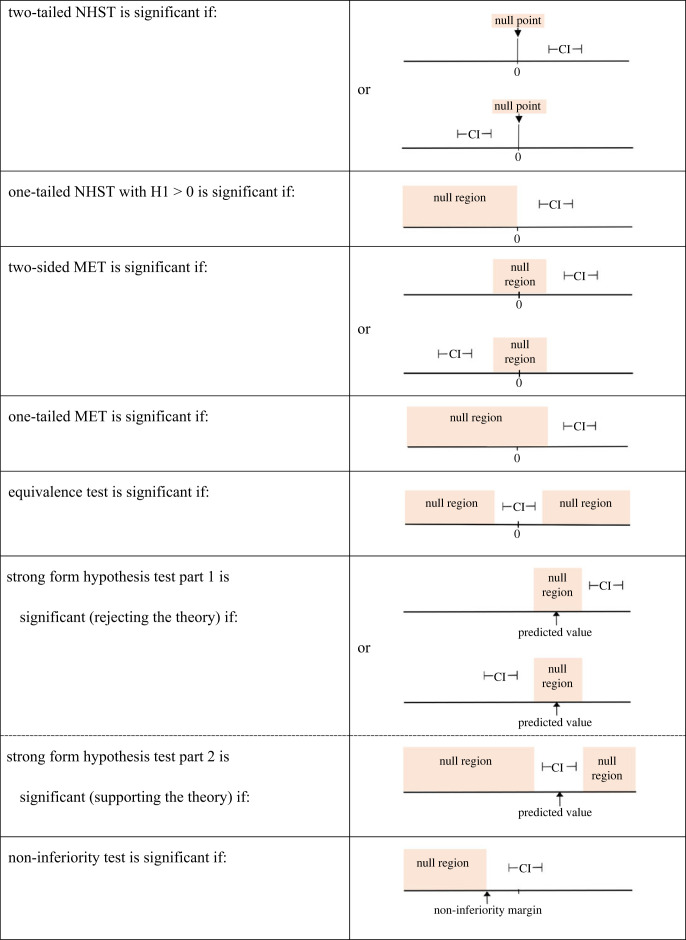
Each test in the proposed framework can be conducted by simply determining if the confidence interval based on the data obtained is entirely outside of the prespecified null region(s).

In figure 2, examples of statistically significant results are shown for each statistical test. The CI represents the confidence interval based on an observed effect, and in the examples in figure 2 the tests are significant because the CI is completely outside of the null region. One-sided MET is analogous to one-sided NHST except that the null region extends beyond 0 to the weakest effect still considered meaningful. If the CI for the observed effect is even partially within the null region, then the test would be non-significant and the null hypothesis will be retained.

### 2.2. Specifying the null region(s)

Specifying the null region(s) plays a critical role for hypothesis testing in the proposed framework. Among clinical trials researchers, reporting of null regions has become a standard research practice. The key for successful non-inferiority testing in clinical trials is transparency: null regions are explicitly stated and clearly presented and easy to find in the text. For example, a 2020 article published in *Lancet Oncology* states the null region (referred to as the ‘non-inferiority margin, or NI margin’) in its online abstract: ‘The overall survival NI margin was set to be HR = 1.11’ [33, p.1620]. The body of the article provides more information in a 410-word paragraph dedicated to the justification and discussion of the non-inferiority margin used in the analysis, vetted through the review process the article underwent. Because it is stated prominently even in the online summary, it makes it clear that interpretations of the results as being ‘significant’ are contingent on the null region. Such transparency, combined with the publication of detailed guidelines for setting the null region (e.g. [29,34–37]), is what makes non-inferiority testing a widely accepted tool in clinical trials research, addressing the temptation by researchers to use the specification of null regions to take advantage of ‘researcher degrees of freedom’ [38].

Outside of clinical trials research, few scientific communities have established norms or traditions concerning the specification and communication of null regions. Thus, an important challenge for any field of study (or scientific journal) is developing practices and standards for specifying them. Below, as a guide, we summarize a number of approaches that could be used to specify meaningful null regions. They are not meant to be exhaustive; there may be other approaches to specifying null regions that have not yet been suggested and that are more appropriate for a given research question. We encourage each scientific discipline to develop their own guidelines and practices most applicable to their research question. Regardless of the specific guideline, or whether or not a guideline is available, we recommend that null regions be specified and pre-registered prior to data collection/analysis whenever possible to reduce the impact of researcher degrees of freedom [38].

#### 2.2.1. Any non-zero true effect is meaningful

In some circumstances, *any* non-zero true effect might be meaningful. Such a case might arise if any non-zero effect would be practically (e.g. a potential intervention has essentially no cost) or theoretically meaningful (e.g. just the presence of an effect regardless of size is an important discovery). Researchers, however, should *explicitly* state and justify why any true non-zero effect would be meaningful. For example, Albert Einstein's theory of general relativity predicted that light would be bent as it passes by enormously massive objects such as the sun. Because few (or, possibly no) other theories predicted this, testing, and rejecting, the point null hypothesis of absolutely no bending helped rule out many (or all) existing theories.

#### 2.2.2. Utility analysis

Utility analysis involves comparing the costs of an intervention against its benefits measured in ‘utility based units' [39]. This utility is often described in terms of financial consequences [40], or in terms of patient outcomes such as quality adjusted life years [39]. Regardless of the units used, the null region(s) are set with regard to the cost and benefit (e.g. setting a boundary of the null region where cost and benefit ‘break even’). Utility analysis can be considered as an option for setting the null region(s) when there are both measurable costs and benefits which can be weighed against one another.

#### 2.2.3. Clinical significance

In the context of clinical intervention research, traditional statistical significance testing has long been criticized for its failure to accurately reflect clinically meaningful change, because a statistically significant finding is not necessarily clinically meaningful (e.g. [41]). Jacobson and colleagues [42] first proposed clinical significance in the context of clinical practice as a function of whether a client has moved from the dysfunctional to functional range on the assessment used to track change during treatment. Similarly, in medicine, the concept of *minimal clinically important difference* (MCID) [43] has been proposed and clinical researchers are urged to make active use of it (e.g. [44,45]; see [46] for an extensive discussion of related terms, such as minimal important difference (MID), minimally important change (MIC), clinically important differences (CID), and smallest real difference (SRD)).

#### 2.2.4. Practical significance

Clinical significance can be thought of as an evolution of a more general concept of practical significance [47]. For example, the upper bound of the null region for MET can be the smallest true effect size that would be of potential utility in *practice* or in the real world [48]. Example 3 of electronic supplementary material S3 illustrates a rationale for selecting a null region based on practical significance, and how it can be reported in publications.

#### 2.2.5. Ambient noise level

For many research questions, for example in research where there is no obvious cost to weigh against outcomes, the approaches above may not be feasible or appropriate in specifying the null region. Under such circumstances, one possible basis for specifying the null region is an estimate of study-to-study variations in the effect of interest, based on a meta-analysis of relevant studies (e.g. [49]). Another possible basis is using the theoretically predicted minimum amount of noise, such as the photon noise in a photoreceptor due to the quantum nature of photons [50], or cosmic microwave background radiation, theorized to reflect the Big Bang [51].

When there is no relevant literature, a method of last resort may be to consider a research domain-specific estimate of ‘ambient noise level’ [52] (also called the ‘crud estimate’ [53]). This refers to the fact that even in extremely large samples, non-zero correlations exist between variables with no obvious relation to each other (e.g. interest in woodworking and birth order). For example, because of associations due to historical accidents (e.g. [54]) rather than to any intrinsic or theoretically meaningful relationship, the correlation between two variables that are conceptually not related at all is unlikely to be zero. Similarly, in an experiment, even if the theoretical variable intended to be manipulated has no true effect at all, an actual manipulation may have a non-zero effect because it is virtually impossible to manipulate only the theoretical variable of interest and no other variables (i.e. it is impossible to eliminate all confounds).

The ambient noise level has been estimated in several studies by examining the average correlation coefficients in very large correlation matrices [55–57]. The precise magnitude of the ambient noise level remains an open question, and it likely varies in size from discipline to discipline. Future research is necessary to determine if it is possible to achieve greater consensus on general and domain-specific estimates of the ambient noise level and best practices for accounting for it (see [53] for suggestions of future directions). Nevertheless, when there are no other bases for setting the null regions, using the ambient noise level to inform null regions can reduce the likelihood of labelling relationships that reflect historical accidents as theoretically meaningful.

### 2.3. *A priori* specification of the null region(s)

We suggest that null regions be specified and pre-registered prior to data collection/analysis whenever possible. Without specifying the null region prior to data analysis, researchers may be more likely to fall into committing *post hoc* rationalizations, or *HARKing (hypothesizing after the results are known*) [58]. Researchers can use pre-registration as a way to show that their null regions were specified *a priori*.

### 2.4. Considerations for editors and reviewers

Although we hope that researchers will be unbiased in specifying null regions, it is important to consider the incentives that affect researchers. For example, some researchers may be motivated to specify an excessively narrow null region(s) for MET, out of concern that setting a wide null region would make it difficult to reject the null hypothesis. For this reason, editors and reviewers should consider the appropriateness of the null regions chosen by researchers. Such a process has become increasingly established in clinical trial research, for the purpose of non-inferiority testing. Other fields may benefit from examining such practices and incorporating the ones that are appropriate for them. In addition, we believe there are other potential mechanisms that can be used to address this issue (for an example, see electronic supplementary material S4), such that the balance of the authors' and reviewers’ incentives, in addition to their commitment to the fundamental goals of science, will result in constraining the proposed null regions within a reasonable range. This process will also facilitate discourse about effect sizes as well as transparency in the research process.

## 3. Reporting findings

In electronic supplementary material 3, we have provided three examples of how the tests in the proposed framework may be used in research and how they may be reported in publications. These examples demonstrate that they can be reported concisely, and they can be used in addition to, rather than instead of, the results of traditional NHST.

### 3.1. Registered reports

The proposed framework solves an important challenge for the promising new publication mechanism called the *registered report*. Registered reports are a publishing format which conduct peer review prior to data collection. Publication of the results is provisionally accepted as long as the authors closely adhere to the registered methodology [59]. It minimizes a variety of questionable research practices, such as publication bias and selective reporting of ‘significant’ or hypothesis-confirming results, while making it possible for reviewers to help authors to improve the proposed research while they can still make changes [60].

One important question, however, has remained: How does one define that a prediction has been confirmed? Considering predictions as confirmed when there is a statistically significant effect using NHST is problematic because as long as the true effect is not exactly zero, traditional two-tailed NHST statistical significance is all but guaranteed as the sample size increases. Considering that the prediction is confirmed when the *observed* effect was larger than the pre-registered minimum predicted effect is also problematic. This is because the likelihood of such an outcome is higher, rather than lower, with a smaller sample size (hence greater standard error), in effect incentivizing researchers to opt for smaller sample sizes.

The proposed framework avoids both of these problems by encouraging researchers to state the predicted outcome in terms of population values, and test if the findings support the prediction. For example, suppose in a registered report authors predicted that a new housing policy would reduce the number of people experiencing homelessness in the population by at least 10%. In this instance, MET is the most appropriate test. First, unlike NHST, unless the reduction observed in a study is greater than 10%, increasing the sample size will not increase the likelihood of the results being significant by MET, as described in the next section. Smaller sample size and the resultant larger standard error will increase the probability of observing reductions greater than 10% by chance, but because MET takes the larger standard error into account, the false positive rate (i.e. Type I error) will be capped at the chosen level of *α* (e.g. 0.05). For an example of MET in a registered report, see [61].

## 4. Implications for sample size

Open science practices increasingly recognize the importance of adequately large sample size. At the same time, when using traditional NHST, increasing the sample size will make it more likely to reject the NHST null hypothesis as long as the true effect is not exactly 0. Results of studies using exceptionally large sample sizes can become unintentionally misleading if one *only* considers whether the traditional NHST *p*-value is statistically significant. Unlike traditional NHST, however, when using the *null regions framework* described above, larger sample sizes do not result in misleading conclusions. If the observed effect falls within the null region(s), it will *never* be significant by any of the tests in this framework, regardless of sample size. This is because regardless of the sample size, hence regardless of its width, a confidence interval will always contain the observed value. Thus if the observed value is within a null region, the confidence interval can never be entirely outside of the null region. On the other hand, if the observed effect falls outside of the null region(s), increasing sample size, hence narrower confidence interval, makes it more likely that the confidence interval around the observed effect is entirely outside of the null region(s). Another way to describe the consequence of increasing sample size is that it would decrease the likelihood that the results are inconclusive (e.g. when the bounds of the confidence interval are partially in and partially outside of the null region).

## 5. Reporting confidence intervals

Confidence intervals are highly informative, and we encourage reporting them whenever possible, while making sure they are interpreted accurately (e.g. not as if they can answer a Baysian question about the likelihood of the population parameter given observations) and not just only for the purpose of seeing if they include 0, which is no different from the traditional NHST. We hope that the framework we presented here will increase researchers' focus on confidence intervals and facilitate the interpretation of the confidence intervals and in making inferences based on them. In the past, researchers often relied on intuitive, and often *post hoc*, interpretations of confidence intervals to support such inferences. We hope our framework makes it possible to do so on a more formal basis by comparing the confidence interval against the null region(s) that are specified based on well-reasoned rationale, reflecting the research questions being asked and the nature of the phenomena being studied.

## 6. Conclusion

With the *null regions framework*, researchers can test a theory more comprehensively than with traditional NHST alone, going beyond simply determining if they can reject a ‘straw man’ null hypothesis of no effect at all. They can test if the observed data allow researchers to infer that the true effect is strong enough to matter (MET), different enough from the theoretically predicted value suggesting that we consider ruling out the theory (Part 1 of strong form hypothesis testing), or close enough to the theoretical prediction, suggesting that we retain the theory (Part 2 of strong form hypothesis testing), or if a new treatment is not meaningfully worse than a current treatment with serious side effects (non-inferiority test). It can provide a strong basis for inferring that the effect is too small to matter (equivalence test), facilitating publication of ‘null’ results and mitigating the ‘file drawer’ problem in the publication process (a bias against publishing findings that are not statistically significant). Most importantly, with greater quantity and quality of data, these tests make it more difficult to defend sub-optimal theories, in contrast to traditional NHST for which the opposite is the case. And it provides a language for stating the inference very concisely (e.g. ‘significant by MET’), for example in abstracts where brevity is paramount, while at the same time requiring researchers to explicitly spell out the basis for the inference in the main body of the paper (i.e. confidence intervals and the range of population values pre-declared as meaningful and appropriate for the research question). It supports the emerging practice of registered reports by providing a much-needed and a more meaningful way of stating the criterion for the observed effect to be considered as ‘successfully supporting the prediction’. The rationale underlying all these tests, and the procedure for conducting the tests, can be conceptualized in a common framework built around the concept of null region(s), visually illustrated in figure 1 (the common rationale) and figure 2 (the common procedure for testing). In sum, we believe that the framework proposed here can lead to improvements in the quality of data-based inferences in scientific research.

## Data Availability

Supplementary material is available online [62].

## Footnotes

1 Serlin & Lapsley [6] took this argument a step further, writing in the abstract, ‘because the psychological null hypothesis is *always false*, increases in precision in psychology always lead to a weaker test of a theory, whereas the converse is true in physics’ (p. 73; emphasis added).

2 Rather, a more accurate interpretation, without slipping into a Bayesian view of probability, is that 95% confidence intervals computed in each sample are expected to contain the true population parameter 95% of the time in the long run [14,15].
